# Recent progress in atomistic modeling of light-harvesting complexes: a mini review

**DOI:** 10.1007/s11120-022-00969-w

**Published:** 2022-10-07

**Authors:** Sayan Maity, Ulrich Kleinekathöfer

**Affiliations:** Department of Physics and Earth Sciences, Jacobs University Bremen, Campus Ring 1, 28759 Bremen, Germany

**Keywords:** Multiscale modeling, Light-harvesting complexes, QM/MM simulations, Excited state calculations, Spectral densities, Exciton dynamics

## Abstract

In this mini review, we focus on recent advances in the atomistic modeling of biological light-harvesting (LH) complexes. Because of their size and sophisticated electronic structures, multiscale methods are required to investigate the dynamical and spectroscopic properties of such complexes. The excitation energies, in this context also known as site energies, excitonic couplings, and spectral densities are key quantities which usually need to be extracted to be able to determine the exciton dynamics and spectroscopic properties. The recently developed multiscale approach based on the numerically efficient density functional tight-binding framework followed by excited state calculations has been shown to be superior to the scheme based on pure classical molecular dynamics simulations. The enhanced approach, which improves the description of the internal vibrational dynamics of the pigment molecules, yields spectral densities in good agreement with the experimental counterparts for various bacterial and plant LH systems. Here, we provide a brief overview of those results and described the theoretical foundation of the multiscale protocol.

## Introduction

During the photosynthesis process, light-harvesting (LH) protein–pigment complexes of plants, bacteria, and algae play a key role in the conversion of solar energy into sustainable forms of chemical energy. Chlorophyll (Chl), bacterio-chlorophyll (BChl), and bilin molecules are the major pigments present in those complexes that absorb sunlight. The excitation energy is subsequently transferred within the pigment network via excitation energy transfer processes (Cogdell et al. 2006; Blankenship et al. 2011; Blankenship 2014). The target of these LH complexes is to transport the solar energy in the form of excitons to reaction centers where the electron–hole charge separation takes place for further processing in photosynthesis.

Photosynthesis can be categorized as oxygenic and anoxygenic depending on its ability to produce oxygen or not. Oxygenic photosynthesis is a process in which water molecules are oxidized into molecular oxygen and is mainly performed by plants, marine algae such as diatoms, and cyanobacteria. Green sulfur and purple bacteria are known to conduct anoxygenic photosynthesis where a terminal reductant like hydrogen sulfide (H$_{2}$S) is split into a byproduct like sulfur. A general equation of biological photosynthesis (Blankenship 2014) can be given by

1 $$n{\text {CO}}_{2}+2n{\text {H}}{_{2}}{\text {X}} +hv \rightarrow ({\text {CH}}_{2}{\text {O}})_{n} + 2n{\text {X}} + n{\text {H}}_{2}{\text {O}},$$

where H$_{2}$X is a reducing agent such as ${\text {H}}_{2}{\text {O}}$ or ${\text {H}}_{2}{\text {S}}$ used to produce carbohydrates $({\text {CH}}_{2}{\text {O}})_{n}$. In both types of photosynthesis, the solar energy is saved as chemical energy in the form of adenosine triphosphate (ATP) which is then utilized together with the reduced nicotinamide adenine dinucleotide phosphate (NADPH) in the Calvin cycle for the production of carbohydrates (Blankenship 2014). In this process also carbon fixation takes place, i.e., molecular CO$_{2}$ is converted into carbohydrates.

During the energy transfer processes in LH complexes, the excitons are being spread over several pigments and a transfer of the absorbed solar energy in the form of excitons through the pigment network toward a reaction center is necessary for further processing of the energy. In the last decade, an enormous interest in some LH complexes of bacteria has been triggered especially by experimental findings of long-lived quantum coherence at low temperature in the Fenna–Mathew–Olson (FMO) complex of green sulfur bacteria (Engel et al. 2007; Panitchayangkoon et al. 2010). Later on, a similar kind of quantum coherences was also reported for conjugated polymers (Collini and Scholes 2009) and marine algae (Collini et al. 2010) at room temperature. It was initially proposed that those long-lived coherences were purely electronic in nature and that they were caused by correlated fluctuations of the BChl excitation energies (Lee et al. 2007; Wolynes 2009). Theoretical calculations based on classical MD simulations did, however, not find any such correlated fluctuations of the site energies belonging to neighboring pigment molecules (Olbrich et al. 2011a; Shim et al. 2012). Furthermore, recent experiments based on two-dimensional electronic spectroscopy raise some questions concerning the long-lived coherences for LH model systems (Duan et al. 2017a; Thyrhaug et al. 2018; Cao et al. 2020). By now, it is believed that the long-lived oscillations are vibronic or vibrational in nature (Duan et al. 2017b) and are too short-lived to play any significant role in energy transfer processes in LH systems (Cao et al. 2020).

Apart from the LH complexes of bacteria and algae, the LH complexes of plants have of course attracted quite some interest and especially also the photoprotective mechanism. Under excess solar light conditions, LH complexes of plants release excess energy as heat in order to avoid photo-damage. This mechanism is known as non-photochemical quenching (NPQ) of higher plants (Ruban et al. 2007, 2012; Chmeliov et al. 2016). In the NPQ process, an increase of the pH gradient across the thylakoid membrane triggers the switch between the light-harvesting and the quenching modes of the antenna complexes belonging to photosystem II (PSII; Tian et al. 2019; Nicol et al. 2019). Apart from the pH gradient, binding of protein PsbS (PSII subunit S) can also induce conformational changes in the LH complexes leading to an activation of the quenching mechanism (Li et al. 2000; Correa-Galvis et al. 2016; Liguori et al. 2019; Daskalakis et al. 2019a, b). The molecular details and the interplay between different processes are presently an active field of research. In addition to harvest light in a frequency range different from that of the Chl molecules, carotenoid molecules are instrumental in regulating the flow of (excess) energy which eventually can be released as heat (Ruban 2016, 2018; Maity et al. 2019). Based on experimental studies, it is believed that the major light-harvesting complex LHCII and the minor antenna CP29 play the most important role in the PSII complex in order to protect the photosynthetic apparatus from excess solar energy and thus photo-damage (Dall’Osto et al. 2017; Son and Schlau-Cohen 2019; Guardini et al. 2020).

In order to understand the energy transfer dynamics in LH complexes, various exciton transfer models have been built based on crystal structures of plant, bacteria, and algae complexes. In experiment, often two-dimensional spectroscopy has been employed, whereas in most theoretical investigation, classical molecular dynamics (MD) simulations followed by quantum chemistry calculations were carried out. The excitation energies, also known as site energies, excitonic couplings and spectral densities are key parameters which can be extracted in such studies (Olbrich and Kleinekathöfer 2010; Olbrich et al. 2011b, c; Shim et al. 2012; Gao et al. 2013; Cupellini et al. 2016; Sláma et al. 2020). Subsequently, these properties need be utilized as input either in density matrix calculations or in ensemble-average wave-packet dynamics (Aghtar et al. 2012). Spectral densities represent the frequency-dependent system-bath couplings within the framework of open quantum system (May and Kühn 2011) and can be determined via the autocorrelation functions of the site energy fluctuations of the individual pigment molecules. Various approaches have applied to determine the energy gap fluctuations. Among the first ones was the configuration interaction with singles (CIS) scheme (Damjanović et al. 2002) but more popular became the semi-empirical ZINDO/S-CIS scheme (Zerner’s intermediate neglect of differential orbital method with spectroscopic parameters together with configuration interaction using single excitation) and time-dependent density functional theory (TDDFT) calculations. All these theories have to be applied in a quantum mechanics/molecular mechanics (QM/MM) fashion to account for the environments of the pigments and the fluctuations thereof. Because of a high-computational demand of TDDFT calculations when employed along MD trajectories, recently, the time-dependent extension of the density functional tight-binding theory (TD-DFTB) and its long-range corrected (LC) version (Kranz et al. 2017) became popular which has an accuracy similar to that of standard long-range corrected TD-DFT approaches but with a significantly reduced numerical effort (Bold et al. 2020). Moreover, various computational demanding quantum chemical methods such as the second-order coupled cluster (CC2) scheme (Suomivuori et al. 2019), pair natural orbital coupled cluster theory, i.e., a domain-based local pair natural orbital similarity transformed equation of motion-coupled cluster singles and doubles (DLPNO-STEOM-CCSD) (Sirohiwal et al. 2021), the algebraic-diagrammatic construction (ADC) formalism (Suomivuori et al. 2019; Bold et al. 2020), the Bethe–Salpeter equation in the GW approximation (GW/BSE) (Hashemi and Leppert 2021), the multireference configuration interaction-DFT (MRCI-DFT) approach (Maity et al. 2019; List et al. 2013; Bold et al. 2020), and complete active-space SCF (CASSCF) in combination with perturbation theory (CASPT2) (Anda et al. 2019) have been utilized in order to calculate and benchmark the site energies mostly for a geometry-optimized single conformations of pigment molecules. In the present review, we focus on the quantities and functions that were extracted and published by our group for bacterial and plant LH complexes using trajectories based on QM/MM dynamics of the chromophores (Bold et al. 2020; Maity et al. 2020, 2021a, b; Sarngadharan et al. 2022). In the following sections, we will describe the multiscale protocol that was successfully applied to LH complexes in order to calculate site energies, excitonic couplings, and most importantly the so-called spectral densities. Due to the size and the electronic complexity of the Chl molecules of the LH systems, we have employed in our multiscale strategy the numerically efficient DFTB method (Elstner et al. 1998) as the basis of the ground dynamics and the excited states calculations in a QM/MM fashion.

This mini review (for more complete reviews see, e.g., Jang and Mennucci 2018; Segatta et al. 2019; Cignoni et al. 2022), which is certainly incomplete and likely lacks many references of interest, is organized as follows: first several structural properties of LH complexes of bacteria such as LH2 and FMO as well as of plants such as the LHCII and the CP29 complexes are described and discussed. Thereafter, a flowchart of the multiscale strategy is presented and most of the key parameters involved in the scheme are described. Finally, the results regarding several properties like site energies and spectral densities are reviewed while brief conclusions are drawn at the end.

## LH antenna complexes of bacteria and plants

**Fig. 1:**
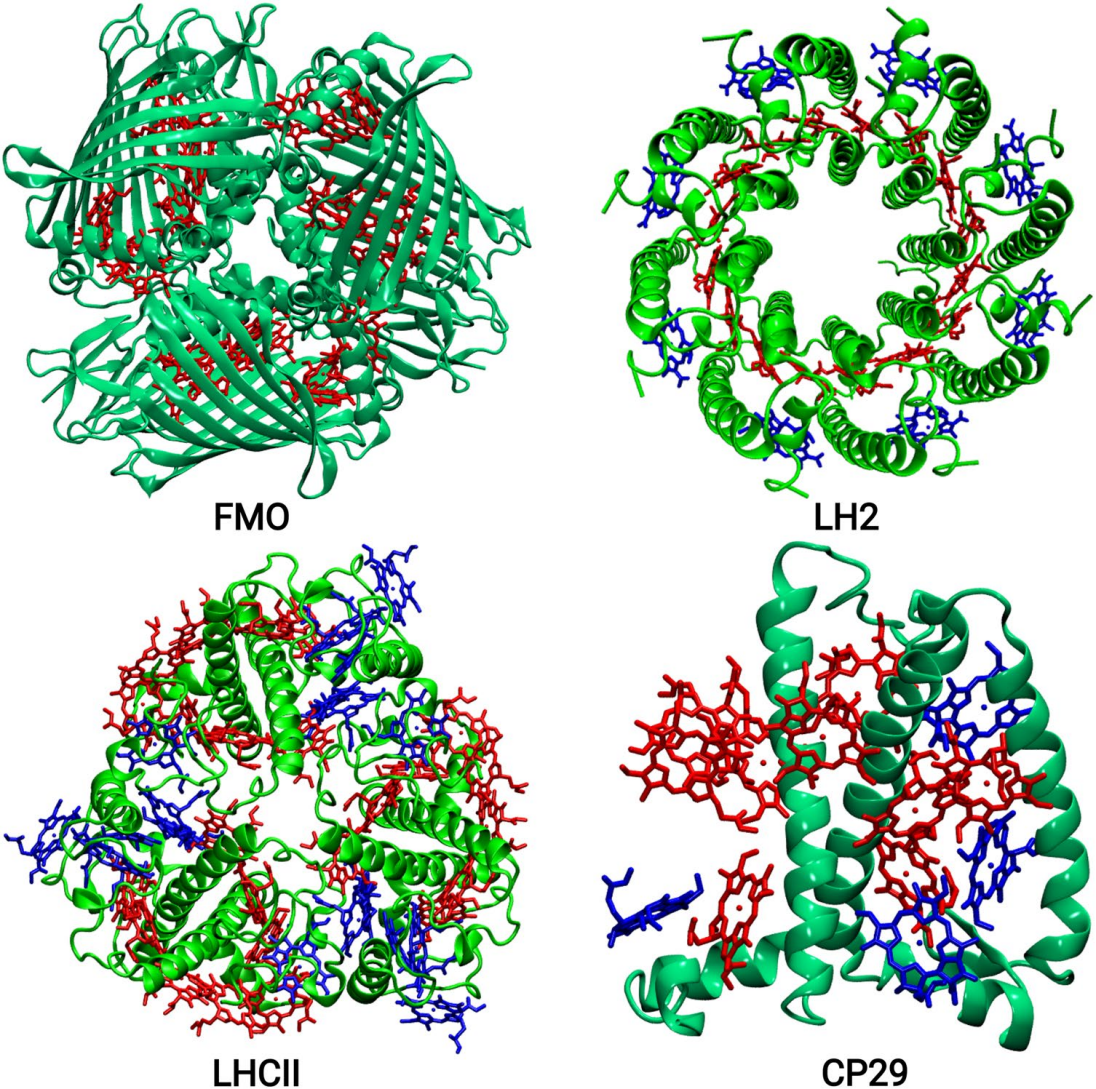
The upper panels depict the bacterial antenna complexes FMO and LH2, whereas the lower panels show the plant antenna complexes LHCII and CP29 with the protein backbone in green cartoon representation. The different types of Chl pigment molecules are highlighted in red and blue

In LH complexes of bacteria, BChl-a molecules are the main pigments involved in harvesting the solar light and in the excitation energy transfer processes. Especially within the last two decades, the FMO and LH2 complexes of bacteria have been extensively studied model systems (see Fig. 1). In case of the FMO complex, the potential of long-lived quantum coherence phenomena at low and ambient temperatures created an immense attention (Engel et al. 2007; Harel and Engel 2012; Duan et al. 2017a; Maiuri et al. 2018; Thyrhaug et al. 2018; Cao et al. 2020). The FMO complex is a water-soluble trimeric LH system of green sulfur bacteria with C$_{3}$ symmetry containing eight BChl in each monomer. In vivo, these proteins are placed in between chlorosomes, i.e., large antenna complex with thousands of pigment molecules, and reaction centers. Their main function is to enable the transport of the excitation energy from the chlorosome to the respective reaction center and thus acts as a kind of excitonic wire. The first X-ray structure for the FMO complex was resolved in 1975 by Fenna and Olson which found seven BChl in each monomer (Fenna and Matthews 1975). Later in 2009, an eighth pigment was found to be present in each monomer which seems to be stably bound only in the trimeric arrangement and can apparently easily be lost in purification processes (Tronrud et al. 2009; Olbrich et al. 2011a). In the year 2007, a wave-like energy transfer in the FMO complex was reported by Engel et al. based on two-dimensional electronic spectroscopy and the coherence time was measured to be up to 1.4 ps at a temperature of 77 K (Engel et al. 2007). An extensive amount of experimental as well as theoretical studies were carried out on this and similar LH model systems with the aim to identify the origin of this long-lived coherence phenomenon (Engel et al. 2007; Harel and Engel 2012; Duan et al. 2017a; Maiuri et al. 2018; Thyrhaug et al. 2018; Cao et al. 2020). It was proposed that correlated site energy fluctuations of the BChl pigments could be reason behind the electronic nature of this phenomenon. Later however, theoretical studies based on classical MD simulations ruled out this assumption (Olbrich et al. 2011a; Shim et al. 2012). Moreover, recent experimental studies lead to the conclusion that the long-lived oscillations are of vibronic or vibrational nature rather than being purely coherences (Duan et al. 2017b; Thyrhaug et al. 2018; Cao et al. 2020).

Other complexes investigated using two-dimensional electronic spectroscopy to look for long-lived coherences were LH2 complexes of purple bacteria (Harel and Engel 2012). They also have been investigated theoretically quite intensively (Hu et al. 2002; Damjanović et al. 2002; Olbrich and Kleinekathöfer 2010; Cupellini et al. 2016). LH2 complexes from purple bacteria are ring-shaped complexes and based on the organism can contain a different number of chromophores, e.g., *Rhodopseudomonas acidophila* and *Rhodospirillum molischianum* include 27 and 24 BChl-a pigment molecules, respectively. Besides the Chl molecules, these LH2 systems also contain carotenoids which act as accessory pigments and are much less studied in experiment and theory partially due to their complex electronic structure. The LH2 systems are split into two rings, one B800 and one B850 ring. These rings are named according to their major absorption wave length in units of nm. The B800 ring contains 9 or 8 BChl pigments, whereas the B850 ring holds 18 or 16 BChl pigments for *R. acidophila* (McDermott et al. 1995) and *R. molischianum* (Koepke et al. 1996), respectively. As can already be seen from these numbers, these systems have either a 8 or 9-fold symmetry depending on the organism. Within the B850 ring, each symmetry unit contains two BChl molecules with a slightly different protein environment leading also to two types of couplings (Alden et al. 1997; Hu et al. 1997). These couplings do, of course, have an influence on the exciton transfer rates between the pigments in the B850 ring (Mirkovic et al. 2017). Since the pigments in the B850 rings are much more closely packed than in the B800 rings, the couplings in the B850 rings are considerably higher than in the B800 rings leading to exciton delocalization in the former rings (Damjanović et al. 2002). For some bacteria, the LH2 complexes together with LH1 systems and F$_{1}$ATPase proteins as well as other proteins are assembled in an organelle termed chromatophore (Singharoy et al. 2019). In such a chromatophore, several LH2 rings surround an LH1 complex, and at the center of these LH1 complexes, a reaction center is present. Recently, an atomistic model of such a chromatophore was constructed containing more than 130 millions atoms (Singharoy et al. 2019). In principle, this model system contains the complete molecular machinery from photon absorption over exciton transfer to the conversion into chemical energy in form of ATP.

In case of plants, Chl-*a* molecules are the main actors in the transfer of the excitation energy to the reaction center, whereas Chl-*b* pigments act as accessory pigments and absorb high-energy sunlight which is subsequently transferred to the Chl-*a* pool. Besides harvesting solar light and producing a significant amount of the oxygen on earth, the LH complexes of higher plants have to protect themselves against too much sunlight. Excess solar energy can lead to an increased lifetime of singlet excited Chls which in turn can trigger a process causing oxidative damage to the pigments. To prevent this damage to happen, an increased pH gradient across the thylakoid membrane is build up in the presence of excess solar energy. Moreover, the binding of PsbS proteins can trigger conformational changes of the protein matrix of the LH complexes in order to release the excess energy as heat. For this NPQ process (Bergantino et al. 2003; Ruban et al. 2007, 2012; Chmeliov et al. 2016; Daskalakis 2018; Liguori et al. 2019), the major antenna complex LHCII and the minor antenna CP29 of PSII have been experimentally identified to play a major role (Dall’Osto et al. 2017; Son and Schlau-Cohen 2019; Guardini et al. 2020). The LHCII complex is present in the periphery of the PSII complex, whereas CP29 acts as a bridge between LHCII and the PSII-core complex. In the presence of excess sunlight, these two complexes are believed to be part of an elegant feedback mechanisms to harmlessly dissipate excess energy (Ruban 2018), while also other proposals for energy quenching exist which are based on fluctuating antenna systems and excitation transfer being modeled in terms of random walks (Chmeliov et al. 2014; Amarnath et al. 2016; Bennett et al. 2018).

The crystal structure of the LHCII system shows that it is a trimeric complex and each monomer contains 14 Chl molecules, i.e., 8 Chl-*a* and 6 Chl-*b* pigments. Moreover, two luteins (Lut), one neoxanthin (Neo), and one violaxanthin (Vio) carotenoid are present in each monomer (Liu et al. 2004). The CP29 complex contains 9 Chl-*a* and 4 Chl-*b* pigments as well as 1 Lut, 1 Neo, and 1 Vio carotenoid (Pan et al. 2011) (see Fig. 1). 

**Fig. 2:**
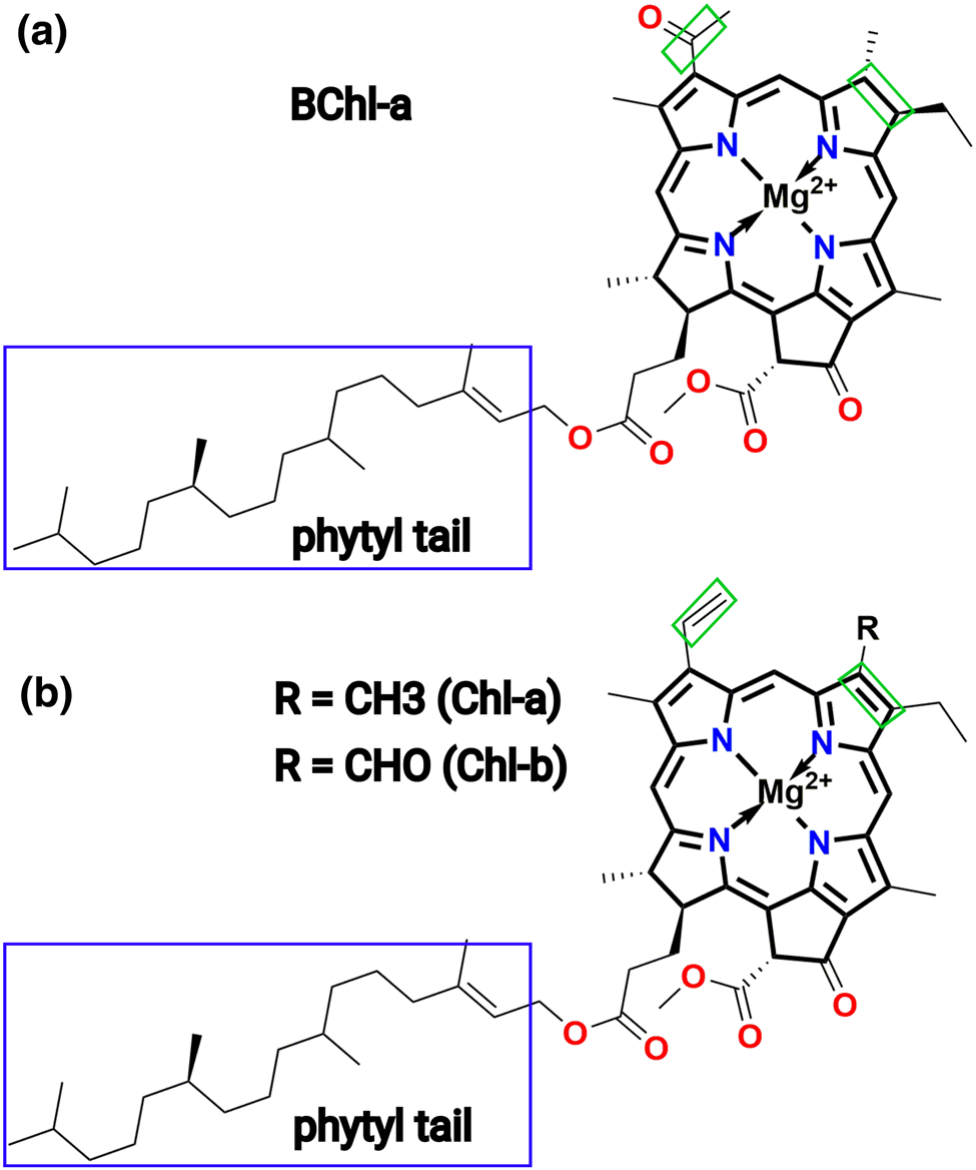
Structural features of **a** BChl and **b** Chl pigments as present in bacteria and plants. The methyl group (R = CH$_{3}$) of the Chl-*a* molecule is replaced by an aldehyde (R = CHO) in the Chl-*b* molecule. The regions highlighted in green in both panels indicate the structural differences between the BChl and Chl molecules

Let us have a brief view on the different Chl types in plants and bacteria since this becomes of interest further below. Chl and BChl molecules are structurally very similar and both pigment have phytyl chain. Moreover, in case of Chl molecules, the Mg-porphyrin ring contains three unsaturated and one saturated pyrrole ring, whereas in BChl, two saturated and two unsaturated pyrrole rings are present in the Mg-porphyrin ring as shown in Fig. 2. Furthermore, in BChl molecules, an acetyl (COCH$_{3}$) group is connected to the Mg-porphyrin ring instead of an ethylene group (CH=CH$_{2}$) in Chl molecules. As a consequence, Chl molecules absorb at a slightly higher energy and are less flexible than BChl molecules which has consequences on the excitation energy transfer processes (Maity et al. 2021b).

## Multiscale description of the LH systems

**Fig. 3:**
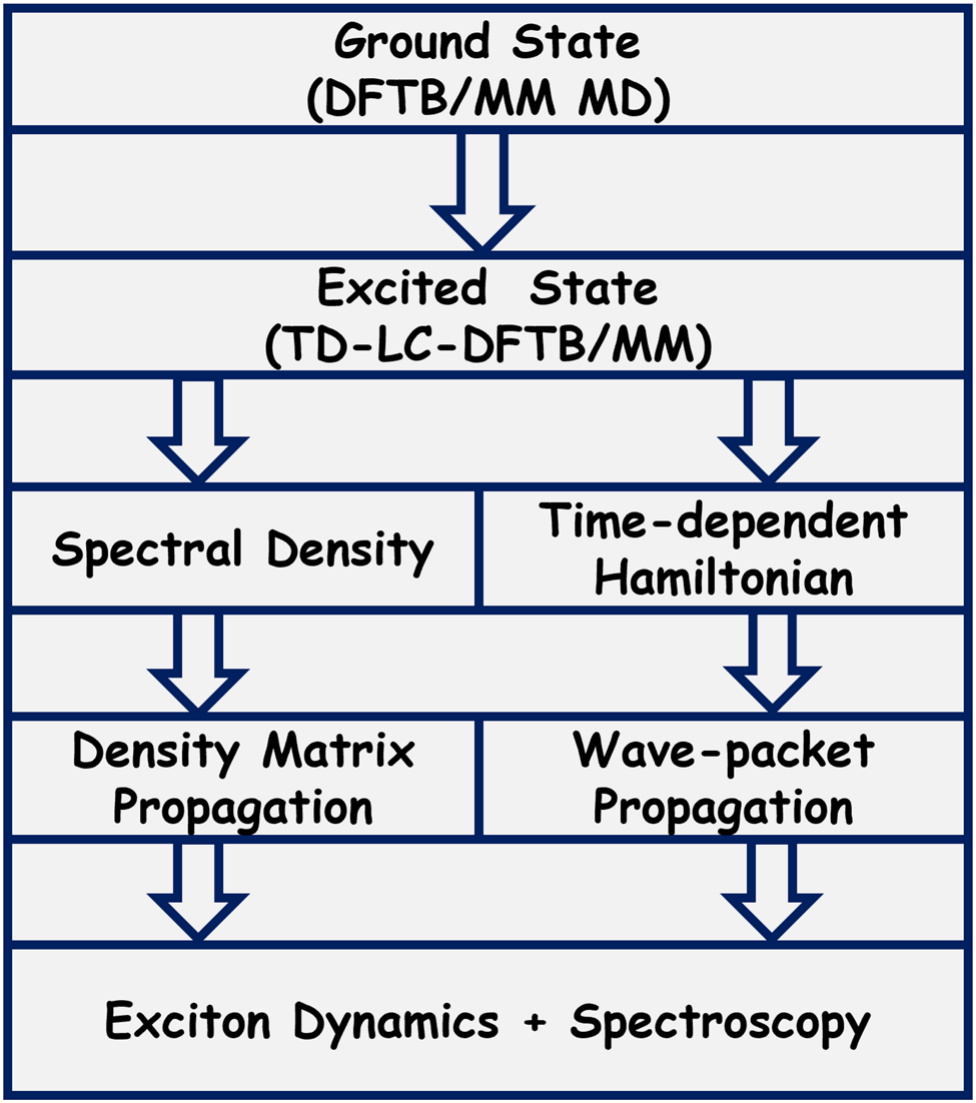
A schematic representation of the multiscale approach employed here to study LH complexes

To study the excitation energy transfer dynamics in LH complexes of bacteria, plants or algae several models have developed within the framework of open quantum systems (Renger et al. 2001; May and Kühn 2011). In this context, mixed quantum-classical ground state dynamics simulations have been performed together with excited state calculations along the trajectory in order to determine the excitonic population dynamics within the pigment network. Over the last years, we mainly followed two strategies to investigate the dynamical properties within LH antenna complexes. On the one hand, a time-average Hamiltonian together with the spectral densities of each pigment can be extracted and utilized as input in density matrix calculations. On the other hand, the time-dependent Hamiltonian can be employed directly to perform ensemble-average wave-packet dynamics. In previous studies especially in our own research group, classical MD simulations were used for the ground state dynamics (Olbrich and Kleinekathöfer 2010; Olbrich et al. 2011c; Aghtar et al. 2014; Aghtar and Kleinekathöfer 2016). It became evident, however, that the force fields are not good enough to describe the internal vibrational modes of the pigment molecules and that these active parts need to be treated on a quantum level. Because of the size and electronic complexity of the pigment molecules, we choose DFTB as the basis of our multiscale scheme for the ground and excited state simulations. A schematic representation of the present multiscale protocol is given in Fig. 3.

The aim of the atomistic simulation is to determine the parameters for a reduced model Hamiltonian. Usually these tight-binding Hamiltonians are restricted to the one-exciton manifold. With this restriction, the Hamiltonian of the Frenkel exciton model is given by

2 $$H_{\text {S}} = \sum _{m} E_{m} |m\rangle \langle m| + \sum _{n \ne m} V_{mn} |n\rangle \langle m|,$$

where $E_{m}$ denotes the excitation energy, i.e., site energy, of pigment *m* and $V_{mm}$ the excitonic coupling between pigments *m* and *n*. The excitonic state $|\alpha \rangle$ can be expressed in terms of the site-local states $|m\rangle$ as

3 $$|\alpha \rangle = \sum _{m} c_{m}^{\alpha } |m\rangle ,$$

and can be obtained by diagonalizing the Hamiltonian. In the following subsections, we briefly explain some of the key ingredients and features of the multiscale scheme and the recent progress in rather accurately describing the exciton dynamics and potentially spectroscopic properties of LH complexes.

### Site energies

The diagonal elements of the Frenkel Hamiltonian as given in Eq. 2 are the excitation energies, i.e., site energies, of the individual pigment molecules. As mentioned above, one way to obtain those site energies is to determine them using quantum chemical approaches along a classical MD trajectory. To this end, mainly the ZINDO/S and TD-DFT (often in a long-range corrected version) methods were utilized in a QM/MM setting and then averaged over time when necessary (Olbrich and Kleinekathöfer 2010; Olbrich et al. 2011b, c; Shim et al. 2012; Gao et al. 2013; Claridge and Troisi 2018; Claridge et al. 2018; Cupellini et al. 2016; Cignoni et al. 2022). TD-DFT schemes are computationally more demanding than the semi-empirical ZINDO/S formalism, and despite some deficiencies, they have been validated and extensively tested for excited state energies of pigment molecules. At the same time, numerous higher-level QM methods for excited state calculations exist but due to their numerical cost, they are usually limited to benchmark calculations for single conformations of pigment molecules. These methods include DFT/MRCI (List et al. 2013; Bold et al. 2020), CASSCF/CASPT2 (Anda et al. 2019), CC2 (Suomivuori et al. 2019), DLPNO-STEOM-CCSD (Sirohiwal et al. 2021), and ADC (Suomivuori et al. 2019; Bold et al. 2020). The recently developed time-depended extension of the long-range corrected DFTB (TD-LC-DFTB) method leads to a numerical formalism which can be employed for many conformations along a trajectory with an accuracy close to that of standard long-range corrected TD-LC-DFT approaches (Bold et al. 2020). For this reason, we have included the TD-LC-DFTB method as the scheme for the excited state calculations into our multiscale strategy as shown in Fig. 3. Since using purely classical MD simulations for the ground state dynamics had clear drawbacks, e.g., in the vibrational dynamics of the pigment molecules, in our recent version of the multiscale scheme, we replaced this classical MD by a DFTB-based MD for the ground state dynamics of pigment molecules in a QM/MM fashion in order to improve the accuracy of the scheme considerably (Maity et al. 2020, 2021a). This change in the multiscale scheme to DFTB/MM MD for the chromophores improved the site energies but also the spectral densities considerably.

**Fig. 4:**
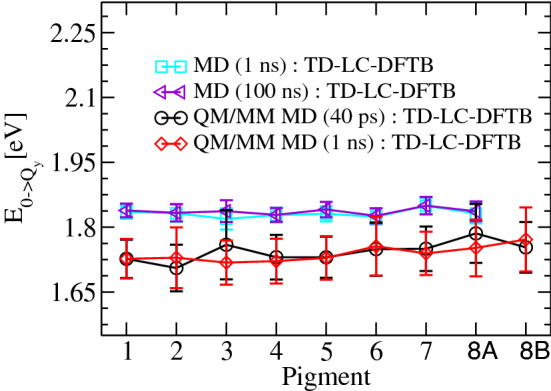
An example of average site energies with the respective fluctuations as error bars for the FMO complex showing excitation energies based on the TD-LC-DFTB method along MD and DFTB/MM MD trajectories of different lengths as published in Maity et al. (2020)

An example of site energies calculated for the FMO complex based on the TD-LC-DFTB method along a DFTB/MM MD trajectory is shown in Fig. 4. Details of these simulations can be found in Maity et al. (2020). These results also show that longer ground state trajectories are necessary to obtain reasonably converged estimates of the average site energies of the individual pigments. We need to mention here that the fluctuations in the MD simulations shown in Fig. 4 are smaller than those in the QM/MM results since in those classical MD simulations bond constraints were employed which are not present in the QM region of the QM/MM simulations (Maity et al. 2020). Moreover, please note that all these site energies are higher than the experimental findings since almost all excited state calculations based on DFT schemes are known to overestimate the excitation energies and band gaps. Within the present QM/MM calculations, only an electrostatic embedding was considered which seems to be a reasonable and an often employed approximation. Moreover, the QM region of the BChl and Chl molecules has been truncated at the C$_{1}$–C$_{2}$ bond of the phytyl tail since the phytyl chain does not significantly contribute to the excitation energies and the truncation of the QM part considerably reduces the computational cost. A more sophisticated QM/MM embedding scheme is often used by the Mennucci group using a polarizable formalism, i.e., the so-called QM/MMPol approach, in which the MM region is described by a polarizable force field (Jurinovich et al. 2015b; Aghtar et al. 2017; Segatta et al. 2019; Cignoni et al. 2022). In this case, the QM and MM regions can mutually polarize each other which is lacking in our electrostatic embedding where only the MM environment can polarize the QM region. Such a polarizable embedding can certainly account more accurate effects of the environment but is computationally more expensive than the more common electrostatic embedding using non-polarizable force fields. Nevertheless, this improved scheme has been applied to biomolecular systems as well (Viani et al. 2014; Loco et al. 2017; Curutchet and Mennucci 2017; Bondanza et al. 2020; Macaluso et al. 2021).

### Excitonic couplings

The second type of ingredients for the system Hamiltonian is the excitonic couplings between pigment molecules, i.e., the off-diagonal terms in the site basis. The excitonic couplings can be seen to consist of two parts: the long-range Coulomb and the short-range exchange contribution. The later term is based on the orbital overlap between the pigment molecules and thus exponentially decays with the distance between the pigments. Due to the pigment distances in LH complexes, the Coulomb term which decays only algebraically with the inter-pigment distance is by far dominating. Thus, the exchange contribution is usually small and often neglected. In a supramolecular approach, however, the full excitonic coupling including exchange and Coulomb parts are included. In such a scheme and for identical donor and acceptor molecules (in the same conformation), the coupling between pigments *m* and *n* is given by Kenny and Kassal (2016) and Bold et al. (2020)

4 $$V_{mn} = \frac{1}{2} (E_{2} - E_{1}),$$

where $E_{1}$ and $E_{2}$ denote the two lowest excitonic energies of the dimer molecule.

**Fig. 5:**
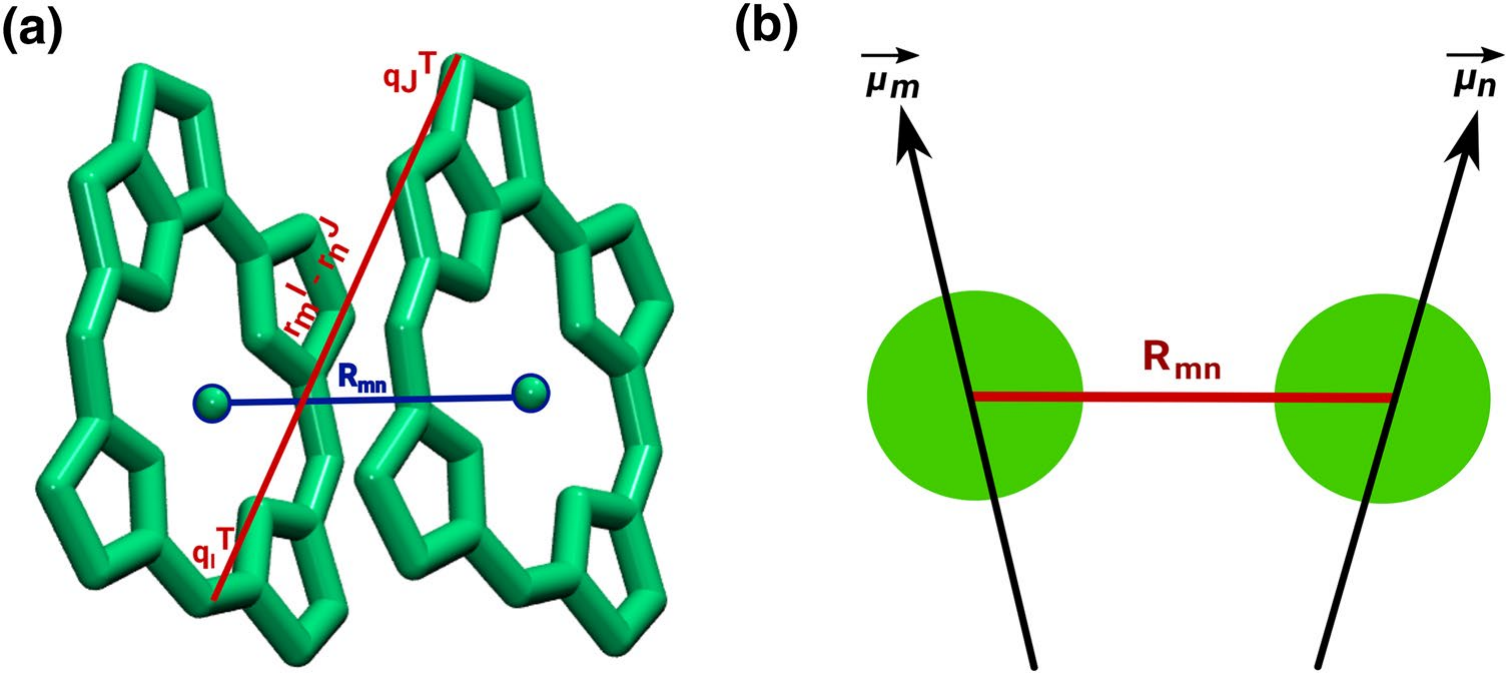
A schematic representation of **a** the TrESP and **b** the point-dipole approximation for a pigment dimer

Without doubt the simplest scheme to determine the Coulomb coupling between two molecules is the point-dipole approximation (PDA) but its range of validity is limited to rather large inter-pigment distances. The coupling between pigments *m* and *n* in the PDA is given by

5 $$V_{mn}^{\text{PDA}}=\frac{1}{4 \pi \epsilon _{0}} \Bigg [\frac{{\mu _{m}} \cdot {\mu _{n}}}{R_{mn}^{3}} - \frac{3 ( {\mu _{m}} \cdot {R_{mn}}) ( {\mu _{n}} \cdot {R_{mn}})}{R_{mn}^{5}} \Bigg ],$$

where $\mathbf {\mu _{m}}$ and $\mathbf {\mu _{n}}$ denote the transition dipole moments of the respective donor and acceptor molecules. A schematic representation of the PDA is shown in Fig. 5. The next simplest scheme is that of extended dipoles in which two transition charges are used per molecule, so that the Coulomb interaction becomes more accurate at intermediate distances (Kenny and Kassal 2016). Using one transition charge per (heavy) atom leads to a scheme called transition charges from electrostatic potential (TrESP) developed by Renger and co-workers (Madjet et al. 2006; Renger et al. 2013). This scheme is basically as accurate as the transition density cube method (Krueger et al. 1998), where the transition charges are located on a Cartesian grid, but the former is numerically much more efficient. Thus, we employed the TrESP formalism in all results shown here. The coupling is given by

6 $$V_{mn} ^{\text{TrESP}} = \frac{f}{4\pi \epsilon _{0}} \sum _{I,J}^{m,n} \frac{q_{l}^{\text {T}} \cdot q_{J}^{\text {T}}}{\mid r_{m}^I - r_{n}^{J} \mid },$$

where the atomistic transition charges $q_{I}^{\text {T}}$ and $q_{J}^{\text {T}}$ are present at atoms *I* and *J* of pigments *m* and *n*, respectively (see also Fig. 5). The transition charges can be determined by the so-called CHELPG (charges from electrostatic potentials using a grid) fitting of the transition density such that the later can be given by a sum of transition charges $\sum q^{\text {T}} \delta (r - R)$. Since TD-DFT-based transition charges usually overestimate the transition dipole moment of pigment molecules with respect to the experimental values, the transition charges are normally rescaled to reproduce the experimental transition dipole values before using them in coupling calculations.

As the space between the pigment molecules is not empty in biological systems, the excitonic couplings are screened. Determining this screening accurately is far from trivial. Mennucci et al. (Curutchet et al. 2009; Curutchet and Mennucci 2017; Aghtar et al. 2017; Cignoni et al. 2022) employed the QM/MMPol approach to determine the environmental effects on the coupling values. So far, this scheme seems the best approach for this purpose but is numerically quite expensive and thus not easily applicable for a large number of pigment pairs along trajectories. Scholes et al. (2007) tried to develop an exponential screening function by fitting a large number of data points. The resultant screening function *f* is given by

7 $$f(R_{mn}) = A \exp (-BR_{mn} + f_{0}),$$

where *A*, *B*, and $f_{0}$ are fitting parameters obtained by comparing to experimental data for several biosystems and have the numerical values 2.68, 0.27, and 0.54, respectively. In case of inter-pigment distances larger than 20 Å, the value of the screening converges to a constant value of 0.54. Alternatively, a constant screening factor as recommended by the Poisson-TrESP approach (Renger and Müh 2012) can be applied in order to capture the environmental effect on the coupling fluctuations. More work in this direction is certainly needed to better include the environmental effects on the coupling values. For example, a screening function has been developed that is not only dependent on the distance of the pigment centers but on all involved atom pairs (Megow et al. 2014).

### Spectral densities

Spectral densities describe the frequency-dependent coupling of a primary system and its environment, also called thermal bath. It has to be clear that in the present case not the whole pigment molecules are included in the primary system but only one mode representing the vertical excitation from ground to the $Q_{y}$ excited state. Thus, large parts of the spectral densities discussed below will be due to internal vibrational modes of the pigment molecules themselves. Denoting the dimensionless coupling parameter for pigment *m* and mode *k* by $c_{m,k}$, the spectral density is given by Weiss (1999), May and Kühn (2011), and Aghtar et al. (2012)

8 $$J_{m}(\omega ) = \frac{1} {2} \sum _{k} \frac{c_{m,k}^{2}}{m_{k} \omega_{k} } \delta (\omega -\omega _{k}).$$

On the experimental side, spectral densities can be extracted from the fluorescence line-narrowing and spectral hole-burning measurements, whereas in our multiscale protocol, we determine it as a half-sided Fourier transformation of the autocorrelation function belonging to the site energy fluctuations (Damjanović et al. 2002; Valleau et al. 2012; Maity et al. 2020) multiplied by a thermal prefactor

9 $$J_{m}(\omega )=\frac{\beta \omega }{\pi } \int \limits _{0}^{\infty } {\text {d}}t\, C_{m}(t) \cos (\omega t).$$

**Fig. 6:**
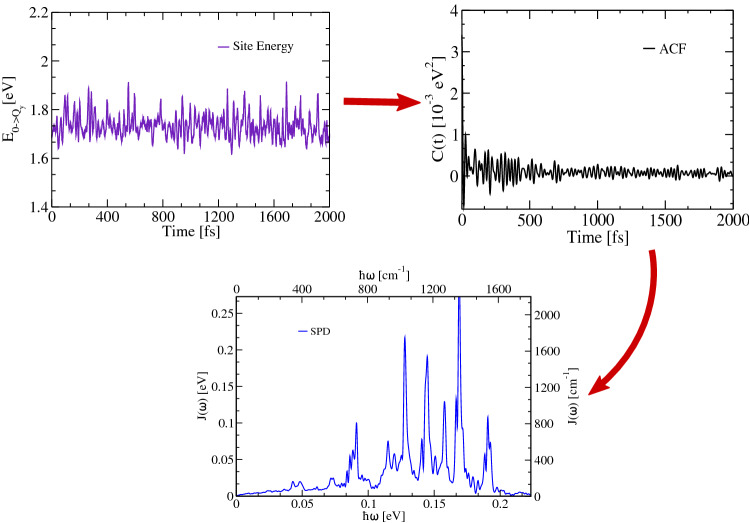
A schematic representation of a spectral density calculation from a autocorrelation function of the site energy fluctuations via the autocorrelation function (ACF)

In this expression, $\beta$ denotes the inverse temperature and C$_{m}(t)$ the site energy autocorrelation function for the corresponding pigment molecule *m*. This autocorrelation function can be determined as

10 $$C_{m}(t_{l}) = \frac{1}{N-l} \sum _{k=1}^{N-l} \Delta E_{m}(t_{l} + t_{k}) \Delta E_{m}(t_{k}) ,$$

where $\Delta E_{m}$ describes the difference between the site energy $E_{m}$ at a certain time point and its time-averaged value, i.e., $\Delta E_{m}(t) = E_{m}(t) - \langle E_{m} \rangle$. Moreover, *N* denotes the number of snapshots present in the respective part of the trajectory. To improve the sampling of the correlation functions and the associate spectral densities along the ground state dynamics, we have employed a windowing technique (Maity et al. 2020). The abrupt start and end of the time series can lead to well-known problems when performing the Fourier transform and can lead to negative peaks in the spectral density. These artifacts can partially be removed by convoluting the time series by a damping function (Cupellini et al. 2018), which however can lead to new issues (Valleau et al. 2012). Thus, we refrained from any damping functions for the results shown here. In the present approach, the negative peaks have simply set to zero since they are unphysical and might lead to erroneous results in the exciton dynamics. We have, however, taken care by tuning the length of the trajectory pieces and the sampling that these problems are reduced to a minimum. Moreover, we have applied the concept of zero padding by extending the correlation functions in order to increase the resolution of the spectral densities. A schematic representation of a spectral density calculation is shown in Fig. 6. Details of a similar scheme are given in Cignoni et al. (2022).

Previous strategies of spectral density calculations based on classical MD trajectories suffered from poor quality ground state geometries used in excited state calculations. This is commonly known as geometry-mismatch problem i.e., the ground state structures based on force fields are not consistent with the excited state quantum calculations (Renger and Müh 2013; Jurinovich et al. 2015a; Padula et al. 2017). Moreover, the internal vibrational dynamics of the Chl and BChl molecules is not well represented by the existing force fields. Attempts like re-parametrizing classical force fields have been made to overcome this problem partially by considering some quantum effects in ground state simulations (Do and Troisi 2015; Claridge et al. 2019). Unfortunately, such approaches still failed to represent the internal vibrational modes accurately leading to inaccurate high-frequency parts of the spectral densities. For this reason, multiscale schemes based on semi-empirical methods (Rosnik and Curutchet 2015) or computationally quite expensive DFT-based approaches (Blau et al. 2018) were employed to overcome this issue. An alternative approach was proposed by Coker and co-workers to calculate the intermolecular and intramolecular contributions of a spectral density separately where the later part is computed using a normal model analysis. In this scheme, the intermolecular part is calculated from pure electrostatic interactions with the environment. Although, this approach has shown quite a good agreement of the resulting spectral density with the experimental counterpart, individual normal mode analyses followed by Huang–Rhys factor calculations within the vertical gradient approximation make this scheme computationally quite demanding (Lee and Coker 2016; Lee et al. 2016; Cignoni et al. 2022). In another approach, Rhee and co-workers constructed a potential energy surface within a QM/MM framework to perform long-time nuclear dynamics and subsequently produced rather accurate ground state conformations in order to use in the excited state calculations (Kim and Rhee 2016; Kim et al. 2018, 2015). Again, however, the construction of such surfaces is computationally expensive for Chl-type molecules since many high-level quantum chemistry calculations are involved. Although this approach provides a good agreement of the spectral density with the experiment finding, some peaks in the high frequency are still problematic (Jang and Mennucci 2018). In addition, completely different approaches exist, e.g., based on normal mode analysis (Renger et al. 2012; Klinger et al. 2020) which have their own advantages but will not be further discussed in this mini review.

**Fig. 7:**
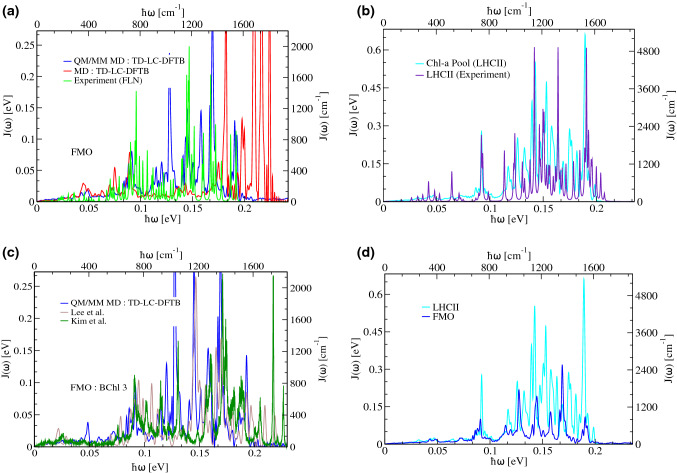
**a** Comparison of average spectral densities for the FMO complex. One spectral density is based on a pure MD ground state dynamics, while the other on a DFTB-based QM/MM MD dynamics. In both cases, the excited states were determined using TD-LC-DFTB. **b** Comparison of a calculated and experimental spectral density for the LHCII complex. **c** For BChl 3 of FMO, the present spectral density is compared to those of Lee and Coker (2016) and Kim et al. (2018). **d** Average spectral densities of the LHCII and FMO complexes which contain Chl and BChl molecules, respectively

Various spectral densities extracted within the present DFTB-based multiscale scheme are shown in Fig. 3. Especially the agreement of theory and experiment for the LHCII complex is remarkable (Maity et al. 2021a), but the same is true for CP29 (Maity et al. 2021b) and CP43 (Sarngadharan et al. 2022) (data not shown). The more surprising is that for the FMO complex, the theoretical results show a main line in the middle of the frequency range in Fig. 7a which is not present in the experimental findings. This fact will need further investigations taking into account that the properties which are compared here are not exactly identical. As can also be seen in Fig. 7a, the results based on the QM/MM ground state dynamics are far superior to those based on a pure MD simulation. Furthermore, we have also compared our results with the spectral densities computed by Lee and Coker (2016) as well as the Rhee group (Kim et al. 2018) in Fig. 7c to get a feeling how the other recent approaches perform. Finally, in Fig. 7d, a spectral density of a BChl-containing complex, here from the FMO complex, is contrasted with that of a system including Chl-*a* molecules, i.e., LHCII in this case, within the same DFTB-based multiscale approach. As can be seen clearly, the rather small structural variations between BChl-*a* and Chl-*a* molecules highlighted in Fig. 2 lead to quite some differences in their internal vibrational dynamics resulting in markedly different spectral densities especially in the high-frequency region.

### Population dynamics

The time-dependent system Hamiltonian can be directly employed to calculate exciton dynamics of the system. In its time-averaged version, it can be employed as in density matrix calculations where one needs to provide spectral densities as additional input functions. Density matrix approaches like the hierarchical equation of motion (HEOM) method are numerically more accurate but computationally demanding for a complex-structured spectral density of LH antenna complexes as shown in Fig. 7. Alternatively, the time-dependent Schrödinger equation needs to be solved using an ensemble-averaged wave-packet-based approach which uses the system Hamiltonian in its time-dependent form. This wave-packet-based approach which is also known as NISE (numerical integration of the Schrödinger equation) is numerically efficient. At the same time, it does not approach the proper thermodynamic limit in general since temperature is not included in these equations but this can be fixed to some extent by introducing correction functions for thermalization (Aghtar et al. 2012; Jansen 2018). Dephasing is, however, treated accurately in this approach. One can indeed show that in certain parameter regimes, converged density matrix results and ensemble-averaged wave-packet outcomes do agree (Aghtar et al. 2012). In the wave-packet calculations, the population of an exciton at pigment site *m* is given by

11 $$P_{m}(t) = |\sum _{\alpha } c_{m}^{\alpha} c_{\alpha }(t)|^{2},$$

where $c_{\alpha }(t)$ is the time-dependent coefficient of exciton wave function in the excitonic basis. In the framework of ensemble averaging, also the population $P_{m}(t)$ has to be averaged over several realizations to obtain a meaningful result.

**Fig. 8:**
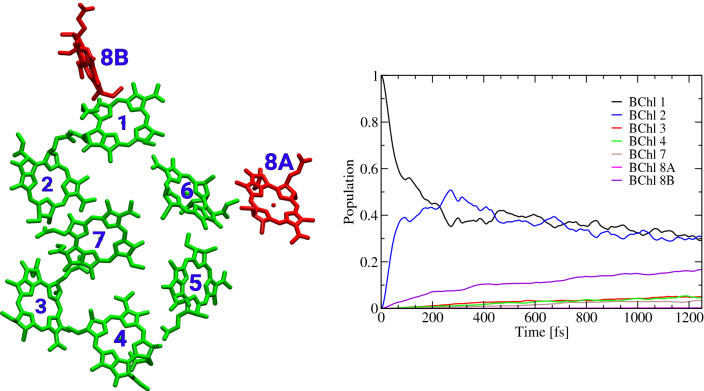
Exciton dynamics in a FMO monomer unit as published in Maity et al. (2020). The right panel shows the BChl network of an FMO monomer together with closely coupled BChl 8 pigment from a neighboring monomer. The left panel shows the exciton dynamics to all other pigments with only BChl 1 being initially excited

As an example, we show in Fig. 8 the exciton dynamics in an FMO monomer. In these calculations, BChl 1 is initially excited and the exciton energy redistribution is monitored up to 1.25 ps. This figure shows that the exciton leaves the initially excited pigment in a roughly exponential manner and is first transferred to BChl 2 and subsequently to the rest of the network. Interestingly, the transfer to the eighth Chl of the neighboring monomer, termed BChl 8B, is rather quick, a point which has been realized already earlier (Olbrich et al. 2011a).

### Absorption spectra

Based on the above-described properties, there are several ways how to determine linear absorption spectra (Schröder et al. 2006; Dinh and Renger 2015; Zuehlsdorff et al. 2019). Here, we employ the often used Redfield-like scheme which makes use of the time-averaged system Hamiltonian together with the site-specific spectral density. Within this approximation, the absorption is given by Novoderezhkin and van Grondelle (2010) and Renger and Müh (2013)

12 $$I(\omega ) \propto \omega \sum _{\alpha} \mid \mu _{\alpha} \mid ^{2} \int \limits _{-\infty }^{\infty } {\text {e}}^{-i(\omega _{\alpha} -\omega )t - g_{\alpha} (t) - {\mid t \mid }/{\tau _{\alpha} }} {\text {d}}t,$$

where $\mu _{\alpha} = \sum _{m} c_{m}^{\alpha } \mu _{m}$ denotes the excitonic transition dipole moments determined from the site basis transition dipole moments $\mu _{m}$. The $\tau _{\alpha}$ denotes the lifetime of the excitonic state $\alpha$ and can be derived using a Redfield-like rate equation. Moreover, $g_{\alpha}$ denotes the excitonic line-shape which can be written as

13 $$g_{\alpha} (t) = \sum _{m} \mid c_{m}^\alpha \mid ^{4}~ g_{m}(t),$$

where the site-dependent line-shape functions $g_{m}$ are determined by the site-dependent spectral densities $J_{m}$ using

14 $$\begin{aligned}&g_{m}(t) = \int \limits _{0}^{\infty } \frac{{\text {d}}\omega }{ \hbar \omega ^{2}} J_{m} (\omega ) \Bigg [(1 - \cos (\omega t)) \coth \bigg (\frac{\hbar \omega }{2 k_{\text {B}} T} \bigg ) \nonumber \\&\quad + i(\sin (\omega t) -\omega t) \Bigg ]. \end{aligned}$$

**Fig. 9:**
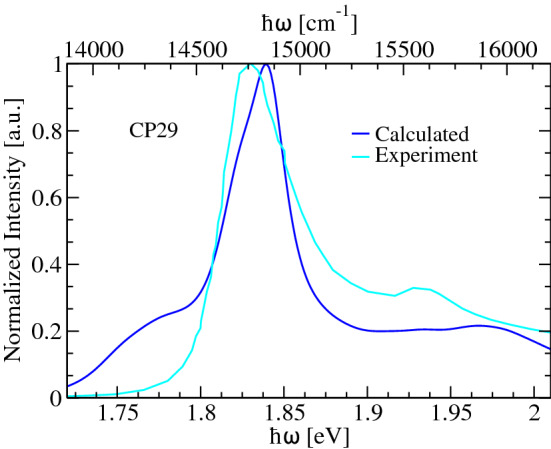
Absorption spectra at 300 K for the CP29 minor antenna complex compared to the experimental findings. The data have been taken from Maity et al. (2021b)

An example of a linear absorption spectrum modeled using the present multiscale approach is shown in Fig. 9. The spectrum belongs to the plant antenna CP29 complex and was already reported earlier (Maity et al. 2021b) . The site energies and transition dipole moments were calculated based on TD-LC-DFTB method along a DFTB/MM MD trajectory. Subsequently, the site-specific spectral densities were extracted from the autocorrelation functions of site energy fluctuations. Moreover, the excitonic couplings were calculated using the TrESP formalism based on a classical MD trajectory. The main peak and the high-frequency vibrational side-band of the calculated spectrum are in good agreement with the experimental counterpart, while we have to mention that the calculated spectrum was shifted such that the peak position agrees. The additional low-frequency shoulder of the calculated spectrum is possibly due to problems with the DFTB calculations of the site energies and/or transition dipole moments. Due to its perturbative nature, the Redfield-like approximation as employed for absorption spectra here can, however, be problematic as was recently shown for the CP43 antenna system (Sarngadharan et al. 2022). In addition, no further broadening due to additional static disorder has been considered during the computation of absorption spectra which would certainly broaden the spectrum to some extent (Cignoni et al. 2022). Nevertheless, the shape of the calculated spectrum is very reasonable, and it is rewarding to see the agreement for the high-energy shoulder which is due to the high-frequency peaks in the spectral densities (Sarngadharan et al. 2022).

## Conclusions and outlook

In this mini review, which is certainly incomplete, biased toward the work of our own research group and likely neglecting many relevant references, we have tried to give a general overview of the challenges which one encounters during the modeling of LH protein–pigment complexes on a molecular level. To this end, we have presented a multiscale strategy that combines DFTB-based ground state MD simulations with TD-LC-DFTB-based excited state calculations and has shown to yield very reasonable results. The key components of this scheme are the site energies, excitonic couplings, and spectral densities which were extracted for various LH antenna complex of bacteria and plants as reported earlier (Maity et al. 2020, 2021a, b; Sarngadharan et al. 2022). Moreover, we have highlighted the problems and the improvements over the previous method based on classical MD ground state dynamics that was unable to describe the high-frequency part of the spectral density accurately. The ingredients that are determined based on the multiscale scheme can be further employed as an input to model the exciton dynamics and spectroscopic properties using many different techniques (Nalbach et al. 2011; Mühlbacher and Kleinekathöfer 2012; Jansen 2021; Varvelo et al. 2021; Kundu and Makri 2022; Bose and Walters 2022).

Despite large progress in the field and despite the remarkable accuracy of the present multiscale scheme, there is still quite some room for improvement from a computational point of view. For example, although DFTB-based ground state and excited state calculations are computationally more efficient than DFT-based approaches, machine learning models can potentially still reduce the numerical cost while increasing the accuracy at the same time. First studies in this direction have been performed already, e.g., in Zaspel et al. (2019), Krämer et al. (2020), Chen et al. (2020), and Westermayr and Marquetand (2020). Moreover, machine learning-based approaches can be further applied for the exciton dynamics calculations which are numerically demanding either in density matrix e.g., HEOM or temperature corrected NISE calculations (Häse et al. 2017). Another improvement could be the “on-the-fly” non-adiabatic dynamics of the exciton dynamics instead of constructing system Hamiltonians as done in the present multiscale protocol. Moreover, there is still the question of how to determine the exciton dynamics in a whole PSII complex or chromatophore. In addition, interesting algae systems exist which so far have investigated far less.
